# Bioethanol production from rice straw by popping pretreatment

**DOI:** 10.1186/1754-6834-6-166

**Published:** 2013-11-29

**Authors:** Seung Gon Wi, In Seong Choi, Kyoung Hyoun Kim, Ho Myeong Kim, Hyeun-Jong Bae

**Affiliations:** 1 Bio-energy Research Center, Chonnam National University, Gwangju 500-757, Republic of Korea; 2 Department of Forest Products and Technology, Chonnam National University, Gwangju 500-757, Republic of Korea; 3 Department of Bioenergy Science and Technology, Chonnam National University, Gwangju 500-757, Republic of Korea

**Keywords:** Popping pretreatment, Rice straw, Bioethanol, Enzymatic hydrolysis, Fermentation

## Abstract

**Background:**

Rice straw has considerable potential as a raw material for bioethanol production. Popping pretreatment of rice straw prior to downstream enzymatic hydrolysis and fermentation was found to increase cellulose to glucose conversion efficiency. The aim of this study was to investigate the influence of popping pretreatment and determine the optimal enzyme loading using a surface response design.

**Results:**

The optimal doses of cellulase and xylanase enzymes were 23 FPU and 62 IU/g biomass, respectively. Using the optimized enzyme condition and popping pretreatment of rice straw (15% substrate loading, w/v), a sugar recovery of 0.567 g/g biomass (glucose; 0.394 g/g) was obtained in 48 h, which was significantly higher than that from untreated rice straw (total sugar recovery; 0.270 g/g biomass). Fermentation of the hydrolyzates by *Saccharomyces cerevisiae* resulted in 0.172 g ethanol/g biomass after 24 h, equivalent to 80.9% of the maximum theoretical yield (based on the amount of glucose in raw material). Changes in the chemical composition and surface area of rice straw were also investigated before and after popping pretreatment. The results showed little or no difference in chemical composition between the pretreated rice straw and the control. However, the surface area of pretreated rice straw increased twofold compared to the control.

**Conclusion:**

Popping pretreatment of rice straw can effectively improve downstream saccharification and fermentation, important for bioethanol production.

## Background

Bioethanol is currently produced primarily from sugar and starch sourced from crops (first-generation biomass) such as sugar cane, wheat and corn, which have a high concentration of sugar [1,2]. However, because these crops are also important food sources bioethanol produced from them can have a significant impact on food prices and food security [2]. In contrast, lignocellulosic biomass, residues from wood or dedicated energy crops (second generation) is an attractive alternative because there is no competition with food and animal feed production, and these materials are also cheaper than first-generation biomass [3,4]. Additionally, the use of lignocellulosic materials as liquid fuels can aid in reducing greenhouse gas emissions [5-7].

Lignocellulosic biomass is the largest source of hexose and pentose sugars, which can be used for the production of bioethanol [8]. Unlike first-generation biomass, in second-generation lignocellulosic substrates cellulose in the cell wall is encased within hemicellulose and lignin matrix, and thus accessibility of cellulose is a major problem in bioethanol production from such sources. Thus, the cost of bio-fuel production is high due to intensive labor and increased processing steps. These economic and technical obstacles must be overcome for efficient and cost effective biological conversion of lignocellulosic biomass into biofuels.

Rice straw is an abundant lignocellulosic waste material in many parts of the world. Rice straw production amounts to approximately 731 million tons per year globally, with distribution in Africa (20.9 million tons), Asia (667.6 million tons), and Europe (3.9 million) [9]. Rice straw is one of the largest biomass feedstocks, and potentially 730 billion liters of bioethanol can be produced per year from the above quantity of available biomass. It is the largest amount from a single biomass feedstock. Presently, high value utilization potential of this biomass remains largely uptapped. Its accumulation in the soil deteriorates the ecosystem via disposal as a waste, and burning in the field air pollution thus which can affect human health [9].

Rice straw consists of cellulose, hemicellulose and lignin. Because cellulose is embedded in a lignin matrix, pretreatment of the lignocellulosic material is needed to enhance the accessibility of this substrate for the conversion of cellulose to glucose. There are a number of biological, physical and chemical technologies available for the pretreatment of lignocellulosic biomass, including use of enzymes, ball milling, steam explosion, acid, alkali, lime and wet oxidation. The slow action of biologically-based pretreatment processes [10], and high cost of ammonia fiber explosion and hot water pretreatment make the processes economically infeasible [11,12]. Therefore, the development of an efficient, cost-effective and environmentally friendly pretreatment method is important [13].

Recently, some new pretreatment technologies have attracted much attention, one of which is popping pretreatment [14-16]. This method is similar to water impregnated steam explosion method, which combines mechanical forces of the sudden explosion with chemical effects from hydrolysis in high temperature water and acetic acid formed from acetyl groups in the biomass. Unlike this method, however, the machine used to undertake popping pretreatment is a very simple system consisting of direct burner and rotary reactor without steam generator. This method offers key advantages over other processes, including significantly lower environmental impact and greater saccharification efficiency over similar methods used conventionally [14], with greater efficiency likely resulting from modification of the substrate that greatly enhances accessibility of desired cell wall components to enzymes. We examined the use of rice straw for ethanol production using the popping pretreatment method developed in our laboratory. Furthermore, the effect of pretreatment on rice straw was tested using downstream processing technologies. Although cellulose enzyme was the main focus of enzymatic saccharification in our study, xylanase was also included with a view to achieving fermentation also xylose with xylose specific yeast in future studies. Additionally, xylanase seemed to have worked synergistically with cellulase.

## Results and discussion

### Chemical composition

The neutral sugar content of rice straw was determined using GC. The composition of straw comprised pentose (24.0%) and hexose (43.7%) sugar, lignin (15.3%) and ash (11.0%) (Table 1). Glucose and xylose were the predominant component sugars in control rice straw, comprising about 41 and 20% of total dry mass, respectively. A small amount of arabinose (3.3%) was present, indicating that the main side chain of the xylan backbone is arabinoxylan. After popping pretreatment, arabinose and xylose contents decreased (Table 1). There was little or no decrease in glucose and lignin contents. The formation of furfural and HMF, byproducts of carbohydrate degradation, was not observed.

**Table 1:** Sugar and lignin compositions of rice straw, expressed as percentages of dry matter

	**Pentose**		**Hexose**				**Total**	**Lignin**		**Ash**
	**Arabinose**	**Xylose**	**Rhamnose**	**Mannose**	**Galactose**	**Glucose**		**Acid insoluble**	**Acid soluble**	
**Control**	3.3 ± 0.2	20.7 ± 0.2	0.3 ± 0.0	0.5 ± 0.2	1.2 ± 0.2	41.7 ± 2.2	67.8 ± 3.2	13.0 ± 0.2	2.3 ± 0.1	11.0 ± 0.5
**Popping**	1.8 ± 0.0^**^	19.3 ± 0.2^**^	0.4 ± 0.0	0.5 ± 0.0	0.9 ± 0.2	41.5 ± 3.6	64.5 ± 4.5	12.2 ± 0.7	2.1 ± 0.1	11.4 ± 0.1

**P < 0.01.

### Characterization of surface area

**Figure 1:**
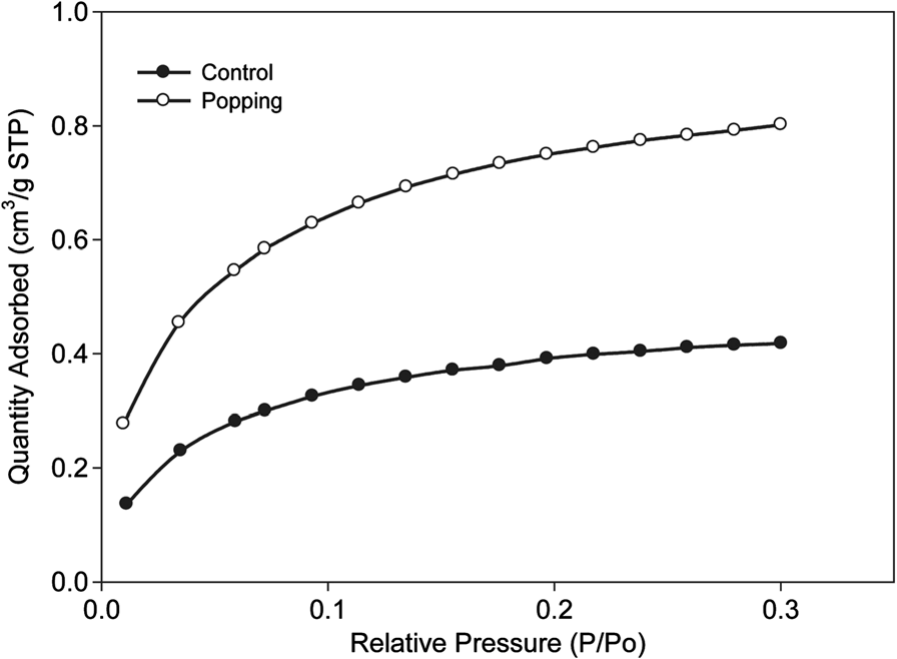
Nitrogen adsorption/desorption isotherms of control and pretreated rice straw powders.

Generally, the Brunauer, Emmett and Teller (BET) equation is used to measure and compare the specific surface areas of a variety of porous materials. The BET surface areas of control and pretreated rice straw were measured by nitrogen adsorption isotherms using a BET surface-area analyzer. The BET surface areas of control and pretreated rice straw were 1.5433 m^2^/g and 2.9346 m^2^/g, respectively (Figure 1). This suggests that the decrease in xylose and arabinose contents (Table 1) that occurred after popping pretreatment resulted in nearly twofold increase in the surface area [17,18].

The morphology of rice straw was studied using FE-SEM (Additional file 1: Figure S1). The surface morphology of pretreated rice straw (Additional file 1: Figure S1d-f) differed markedly from that of control rice straw (Additional file 1: Figure S1a-c). Pretreated rice straw had a rough and porous surface with identifiable micropores (Additional file 1: Figure S1f). The rougher surface and a higher surface area resulting from the removal of hemicelluloses by the popping method enhanced enzymatic hydrolysis, as has generally been considered [17]. These results are consistent with those for rapeseed straw pretreated by the popping method [14].

### Optimization of enzyme loading and saccharification

Enzymatic hydrolysis is a key step in the bioconversion of cellulose to ethanol, and the focus of our research was to improve the yield and rate of enzymatic hydrolysis. Xylanse is known to have a synergetic effect on cellulose hydrolysis by degrading heterogenous xylan polymer that surrounds cellulose microfibrils [14]. Indeed, the supplementation of non-cellulolytic enzymes such as xylanase, pectinase, feruloyl esterase has been known to enhance hydrolysis of lignocellulosic biomass [19]. This is the reason why we used the complex of cellulase and xylanase as a cocktail in this study. The optimization of the enzyme ratio affecting saccharification was carried out following the factorial design of experiments and response surface methodology with factors limited to enzyme loading. We chose the 40 FPU celluase/g biomass as the upper limit, using central point as the median in the range, as there was no further increased in the hydrolysis yield and sugar content above this level of enzyme loading. However, the reason remained unclear. It maybe relates to enzyme absorption on substrates, but this is a speculation. Table 2 shows the experimental matrix for the statistical 2^2^ factorial design. The effects and interaction of cellulase and xylanase were estimated using a test of statistical significance (Additional file 2: Table S1). *P* values > F less than 0.0500 indicate that model terms are significant. Cellulase loading was the most significant variable with a positive effect on enzymatic saccharification. Also, xylanase supplement appeared to enhance the increases in enzymatic hydrolysis yield. Experimental data were fitted to a quadratic model, and the following expression was obtained.

**Table 2:** Experimental matrix for the factorial design and center points

**Run**	**Coded values**		**Reducing sugar (mg/mL)**
	***x***_***1***_	***x***_***2***_	**Experimental**
**1**	-1	1	5.132
**2**	1	-1	5.716
**3**	-1	-1	4.897
**4**	-2	0	2.902
**5**	0	2	5.309
**6**	0	-2	5.088
**7**	0	0	5.807
**8**	0	0	5.667
**9**	2	0	5.444
**10**	1	1	5.601
**11**	0	0	5.717

Response = 5.78 + 0.53∙cellulase + 0.047∙xylanase - 0.088∙cellulase∙xylanase - 0.39·cellulase^2^ - 0.14·xylanase^2^.

**Figure 2:**
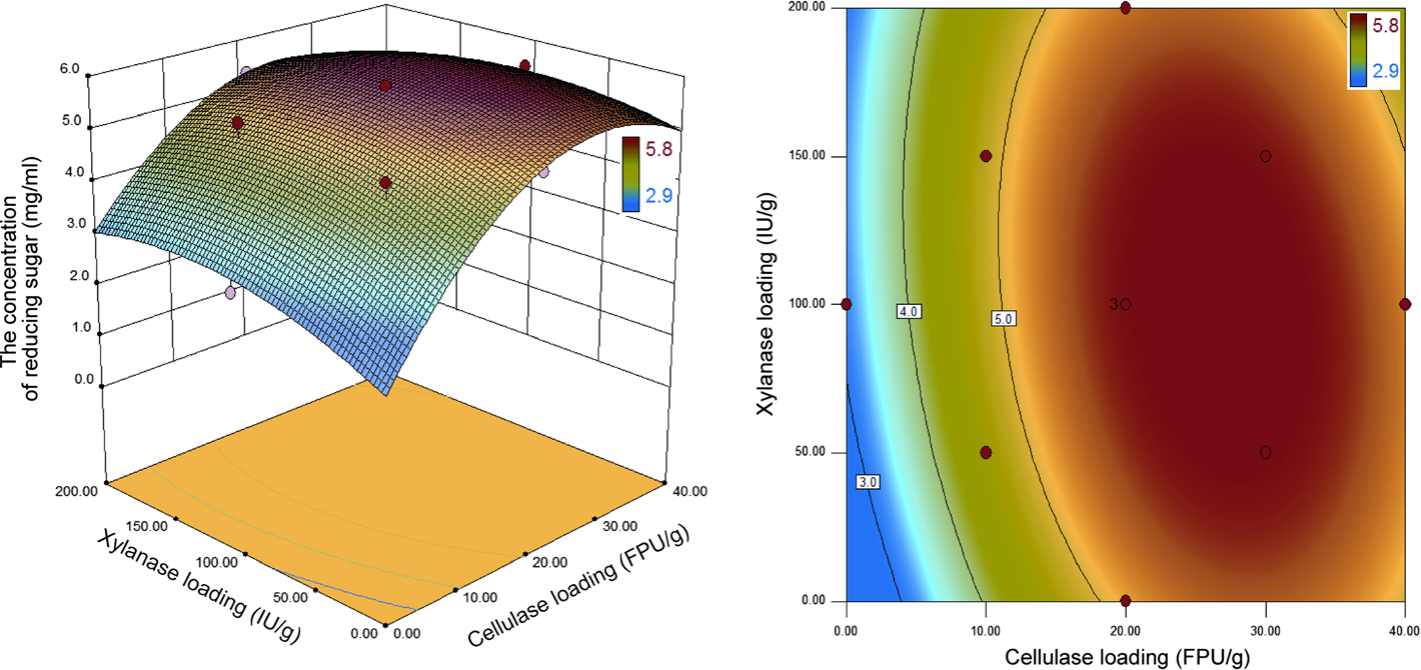
Response surface plot of central composite design for the optimization of the enzymatic hydrolysis of popping-pretreated rice straw.

**Figure 3:**
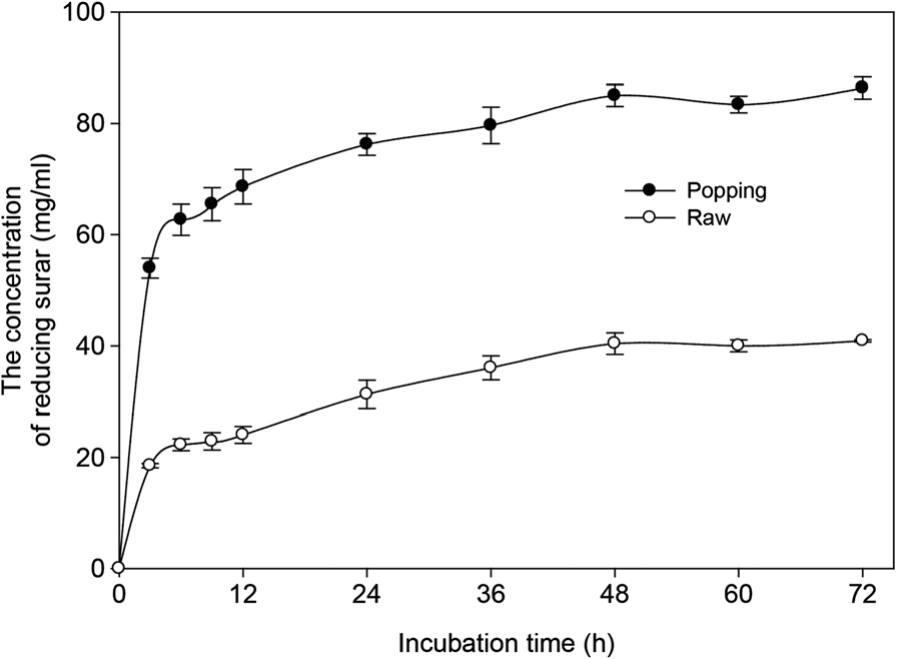
Changes in reducing sugar produced from control and popping-pretreated (at 220°C and 1.96 MPa.) rice straw at 15% DM over time as determined by the DNS method in experiments using an optimized cellulase to xylanase ratio for 72 h of enzymatic hydrolysis.

The relationship between the response and enzymes is visualized by the response surface, while the contour plot gives information about the extent of influence of the parameters (Figure 2). The optimum cellulase to xylase ratio was determined by solving the regression equation; this gave values of 23 FPU cellulase and 62 IU xylanase/g DM. Model verification was performed in three additional trials using the optimized enzyme mixture and was compared to the value predicted by the model. The predicted reducing sugar value was 5.8 mg/mL (Conversion ratio, 86.9%) on the 1% DM loading; the experimental results (85.0 ± 1.6 mg/mL; 85.0%) on the 15% DM loading were similar, indicating that the enzyme mixture validation results were satisfactory (Figure 3). Because enzymes are expensive it was considered that using 1% DM to determine the optimum ratio of enzymes would be a considerable saving on the cost.

### Separate hydrolysis and fermentation (SHF)

**Figure 4:**
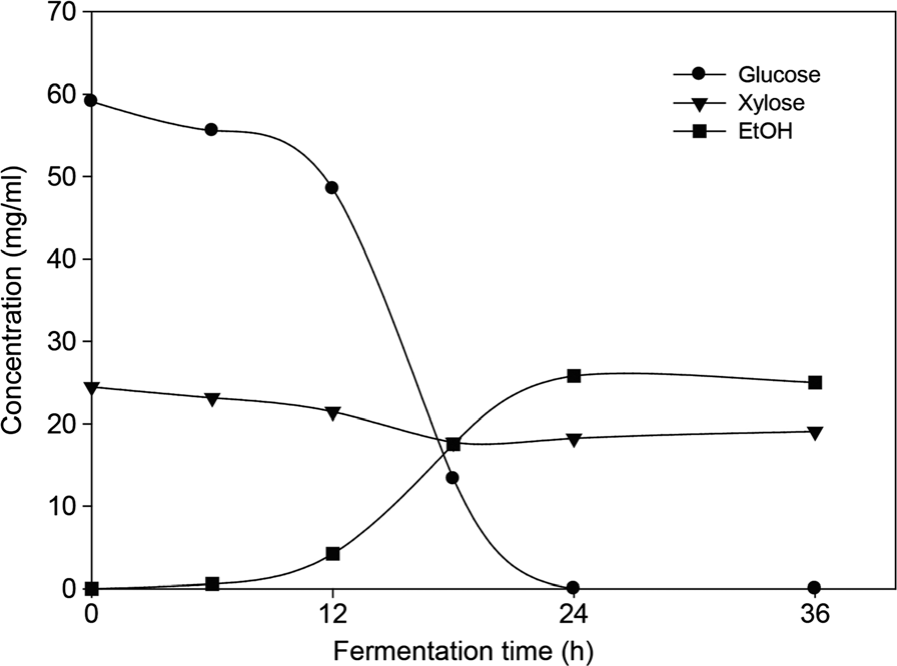
**Time courses of sugar utilization and ethanol production by *S. cerevisiae *** **KCTC 7906 from hydrolyzate by enzyme mixture containing cellulase (23 FPU/g DM) and xylanase (62 IU/g DM) after popping pretreatment.**

To meet economical feasibility in ethanol processes from lignocellulose biomass, high tilter of ethanol must be achieved. For 2^nd^ generation bioethanol fermentation, a high solids loading of the pretreated feedstock close to 30% (w/w) is required to reach the ethanol concentration up to 5% (w/w). However, solid loading above 15% level may not result in greater cellulose conversion in enzymatic hydrolysis or in SSF process, owing to high viscosity and mass transfer [20]. Thus, enzymatic hydrolysis and fermentation experiments were carried out at 15% (w/v) solid loading. Enzymatic hydrolysis of popping-pretreated rice straw resulted in a 3.2 g/L h reducing sugar productivity during the first 24 h, and in a reducing sugar concentration of 85.0 g/L (glucose; 58.5 g/L) after 48 h; corresponding to an 87.2% overall glucose recovery (based on the glucose content in raw material) (Figure 3). In case of rice straw that had not been pretreated, productivity and the final concentration of reducing sugar were 1.3 g/L h and 40.4 g/L (glucose: 22.5 g/L), respectively. In our study, the ethanol concentration in popping pretreated rice straw reached 25.8 g/L, which was based on enzymatic hydrolysis assuming 85.6% fermentation yield within a 24 h period (0.44 g ethanol/g glucose) (Figure 4). The remaining xylose is a pentose sugar that cannot be digested by *S. cerevisiase*[21]. The ethanol yield in this study was ~0.44 g/g, which is in accordance with those reported previously [22-25]. However, the ethanol concentration achieved in this study was not higher than 40 g/L, which is required for feasible distillation. Therefore, in order to achieve higher concentration of ethanol attractive for industrial application, higher rice straw loading is necessary.

### Mass balance

**Figure 5:**
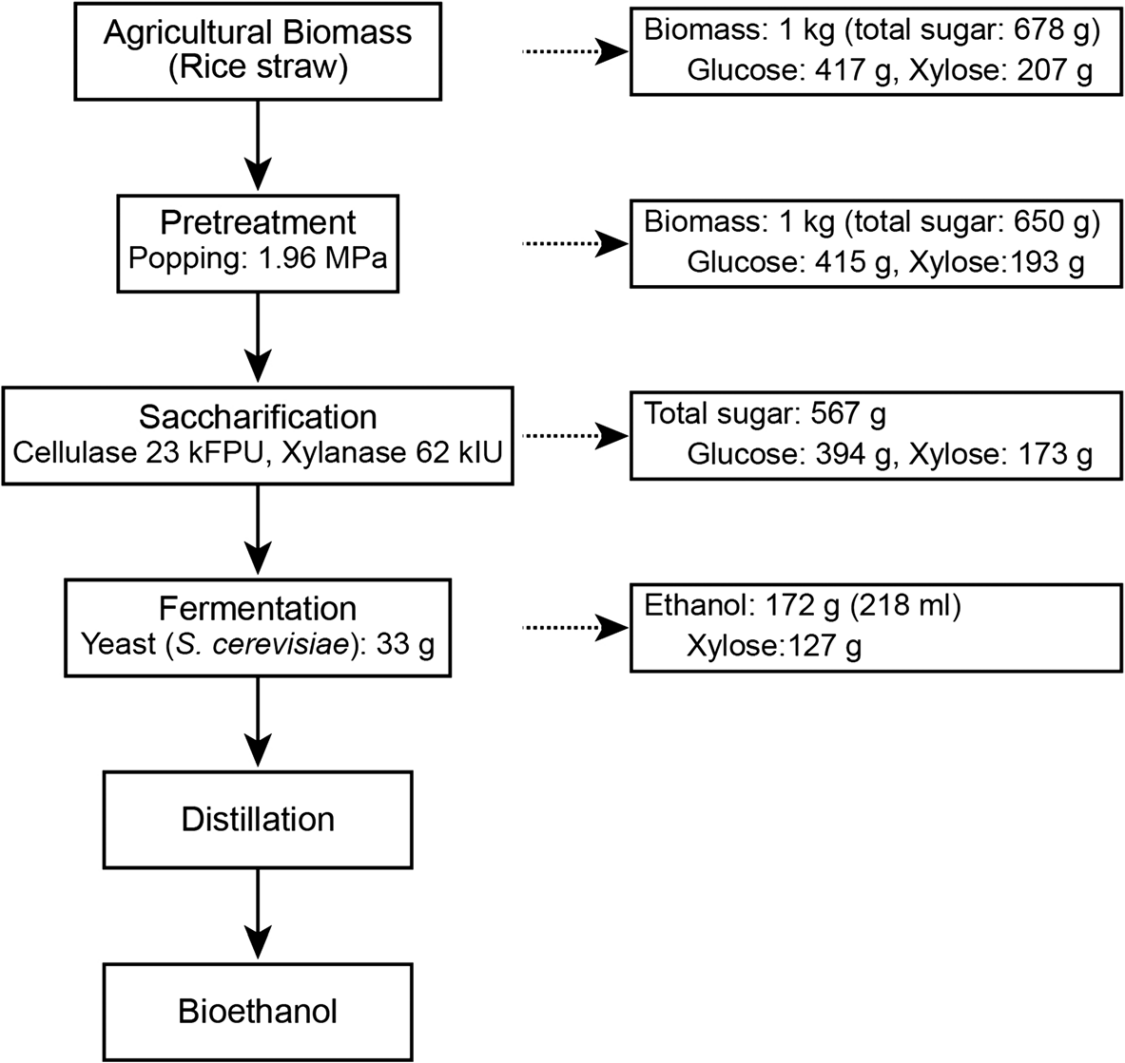
Overall mass balance for the popping pretreatment, enzymatic saccharification and fermentation.

Using composition analyses after each step, we developed an overall mass balance for our operation, including the popping pretreatment, enzymatic hydrolysis, and fermentation steps (Figure 5). Rice straw, after popping pretreatment, can be successfully converted to ethanol by the SHF process. After popping pretreatment, 2 g glucose and 14 g xylose/ 1 kg raw material were decreased. Total sugar after popping pretreatment recovered was 650 g, which is not too far off from the theoretical maximum of 678 g for 1 kg raw material. From the enzymatic hydrolysis step, 394 g of glucose and 173 g of xylose were obtained per 1 kg of pretreated rice straw, when 23 kFPU of cellulase and 62 kIU of xylanase per kg rice straw were used. Fermentation of the hydrolyzates by *Saccharomyces cerevisiae* resulted in 0.172 g ethanol/g biomass after 24 h, equivalent to 80.9% of the maximum theoretical yield (based on the amount of glucose in raw material). The xylose content was fairly high after the popping pretreatment indicated that at the end of the SHF lower ethanol yield mainly resulted from inefficient utilization of xylose by yeast. Future work may also include fermentation of xylose with specific yeast such as *Pichia stipitis*.

## Conclusion

Popping pretreatment of rice straw prior to downstream enzymatic hydrolysis and fermentation increased the efficiency of conversion of cellulose to glucose. The optimal cellulase and xylanase doses for popping pretreated rice straw at 220°C and 1.96 MPa were 23 FPU and 62 IU/g, respectively. Using the optimized enzyme condition and popping pretreatment (15% substrate loading, w/v), sugar recovery of 0.567 g/g biomass (glucose; 0.394 g/g biomass) was achieved in 48 h, which was significantly higher than that obtained from rice straw that had not been pretreated (total sugar recovery; 0.270 g/g biomass). Fermentation of the hydrolyzates with *S. cerevisiae* yielded 0.172 g ethanol/g untreated biomass after 24 h, equivalent to 80.9% of the theoretical yield based on the glucose content of raw material. There was little or no difference between the chemical composition of control and pretreated rice straw. However, the surface area of pretreated rice straw increased twofold over the control. The results obtained suggest that popping pretreatments brought about favorable changes to the substrate, such as increased surface area and larger pore volume, resulting from hemicellulose degradation, which greatly enhanced enzymatic accessibility of the substrate, leading to more efficient hydrolysis of cellulose. Popping pretreatment of rice straw can effectively improve downstream saccharification and fermentation, important for bioethanol production.

## Materials and Methods

### Raw material and popping pretreatment

Rice straw harvested in 2011 was chopped into small pieces of ~2 cm in length with a cutter, ground with a wet-disc mill (particle size: 0.7 ± 0.2 cm) and then kept refrigerated until use. Popping pretreatment was performed in a laboratory-scale cast iron cylindrical reactor with a total volume of 3 L, as described in a previous work [14]. The reactor was filled with 400 g of disc-milled feedstock (moisture content 75%) per batch. That was directly heated with a gas burner at a rate of between 15 and 20°C/min and rapidly open the hatch at 220°C and 1.96 MPa. After popping, the material was recovered in a storage tank and the wet material was cooled to ambient temperature.

### Chemical composition analysis

The ethanol-benzene soluble fraction was determined gravimetrically. Klason lignin, acid-soluble lignin and the ash of raw and pretreated rice straw were analyzed in accordance with TAPPI Standard Methods [26]. Analyses of structural sugars (glucose, xylose, arabinose, mannose, galactose and rhamnose) were conducted using a gas chromatograph [14].

### Enzyme activity

The commercial enzymes used in this study were cellulase (Celluclast 1.5 L, Novozyme) and xylanase (X2753, Sigma). Filter paper unit activity of cellulase was measured in terms of FPU/mL [27]. One filter-paper unit (FPU) was defined as the amount of enzyme required to release 1 μmole of glucose from filter paper per min. Xylanase activity was measured on the basis of xylose released from birch wood xylan as a substrate and was expressed in terms of international units (IU)/mL. One IU was defined as the amount of enzyme required to release 1 μmole of xylose from birch wood xylan per min [28]. The activities of cellulase and xylanase were 79 FPU/mL and 592 IU/mL, respectively.

### Optimization of enzyme mixture

Enzymatic saccharification was conducted at 1% DM (dry matter, w/v) initial substrate loading in a conical tube (50 mL). A sample of pretreated rice straw was soaked in 0.1% (w/v) yeast extract, 0.2% (w/v) peptone and 0.05 M citrate buffer (pH 4.8). Enzymatic hydrolysis was performed at 37°C (the optimal temperature for xylanase) with various enzyme concentrations (0, 10, 20, 30, and 40 FPU cellulase g^-1^ biomass and 0, 50, 100, 150, and 200 IU xylanase g^-1^ biomass) for 48 h. This hydrolytic reaction was followed by measurement of the carbohydrate levels in the hydrolyzates using a DNS assay [29]. Optimization of the cellulase to xylanase ratio was achieved using response-surface methods [30]. In this work, a central composite design was established to study the empirical relationship between the released sugar and enzyme mixtures, namely: *x*_*1*_, cellulase and *x*_*2*_, xylanase (Table 3). Table 3 shows the two variable replicate central composite designs used for fitting of the following quadratic model. Enzymatic conversion yield was calculated as the ratio of glucose released at 48 h divided by the glucose content in pretreated rice straw.

$$y=a+bx_{1}+cx_{2}+dx_{1}\;x_{2}+ex_{1}{}^{2}+fx_{2}{}^{2}$$

**Table 3:** Experimental domain and level distribution used for enzyme ratio optimization

**Variables**	**Coded level**	**Level**				
		**-2**	**-1**	**0**	**+1**	**+2**
**Cellulase (FPU/g biomass)**	*x*_*1*_	0	10	20	30	40
**Xylanase (IU/g biomass)**	*x*_*2*_	0	50	100	150	200

### Separate hydrolysis and fermentation

Enzymatic saccharification was conducted in a 500 mL Erlenmeyer flask with a total working volume of 100 mL at a substrate concentration of 15% DM (w/v) with 0.1% (w/v) yeast extract, 0.2% (w/v) peptone, and 0.05 M citrate buffer (pH 4.8). Reaction flasks were run in triplicate with an enzyme loading of 23 FPU cellulase and 62 IU xylanase/g biomass at 150 rpm for 48 h. The flasks were then stored at 4°C until required fermentation.

For the fermentation with *S. cerevisiae* KCTC 7906, 0.5 g of dry yeast was added as inoculum to 100 mL of hydrolyzates. Fermentation was carried out at 32°C for 48 h with agitation at 150 rpm. All experiments were performed in triplicate, and ethanol yield was calculated on the basis of total glucose content in the pretreated materials by dividing the quantity of ethanol produced by the total amount of glucose.

### High-performance liquid chromatography (HPLC) analysis for liquid phase

During enzymatic hydrolysis and fermentation sugars (glucose and xylose) and ethanol were monitored using HPLC equipped with a refractive index detector (YoungLin Instruments, Anyang, Korea). A Rezex ROA organic acid column (Phenomenex, Torrance, CA) was used for compound identification (300 × 7.8 mm). The temperatures of the column and detector were maintained at 65 and 40°C, respectively, and 5 mM sulfuric acid was added to the mobile phase at a flow rate of 0.6 mL per min.

### Structural characterizations

The surface morphologies of the samples were examined using field-emission scanning electron microscopy (FE-SEM) with a JSM-7500 F (Jeol, Japan) instrument operating at a beam voltage of 3 kV. Prior to observation, each sample was dehydrated with a graded ethanol series and freeze-dried. The external surface of the sample was then sputter-coated with osmium suing a sputter-coater.

### Surface area measurement using a BET

The pore structures of rice straw and its popping pretreated materials were measured using BET nitrogen adsorption-desorption isotherms at -196°C in a surface-area analyzer (ASAP 2020, Micromeritics Co., USA). Prior to determination, the sample (~0.7 g) was degassed for 1.5 h at 110°C under vacuum (5 mmHg) to remove moisture and any other contaminants. The total pore volume was assessed by converting the amount of nitrogen gas adsorbed to the volume (cm^3^/g at STP) of liquid adsorbate, using a single point adsorption (at a relative pressure *circa* 0.99).

## Abbreviations

BET: Brunauer Emmett and Teller; GC: Gas chromatography; DM: Dry matter; FE-SEM: Field emission scanning electron microscopy; FPU: Filter-paper unit; HPLC: high performance liquid chromatography; IU: International unit; S. cerevisiae: *Saccharomyces cerevisiae*; SHF: Separate hydrolysis and fermentation.

## Competing interests

The authors declare that they have no competing interests.

## Authors’ contributions

SGW performed the lab-scale pretreatment, enzymatic hydrolysis, chemical composition analyses including the HPLC and GC, and drafted the manuscript. ISC, KHK and HMK carried out the ethanol fermentation and the determination of ethanol content and help to draft the manuscript. HJB coordinated the study, contributed to the analysis of the results and in the improvement of the manuscript. All authors read and approved the final manuscript.

## Supplementary Material

Additional file 1: Figure S1 FE-SEM photographs of rice straw powders showing the morphology of surface before (a-c) and after popping pretreatment (d-f). Note an increased in micropores (arrows) after popping pretreatment. Click here for file

Additional file 2: Table S1 ANOVA of the adjusted model of the response to enzymatic hydrolysis of pretreated rice straw. Click here for file
