# Supplementary figures and images for: Bioethanol production from rice straw by popping pretreatment
Source: Biotechnol Biofuels. 2013 Nov 29;6:166. doi: 10.1186/1754-6834-6-166 (PMC4176758; doi:10.1186/1754-6834-6-166)

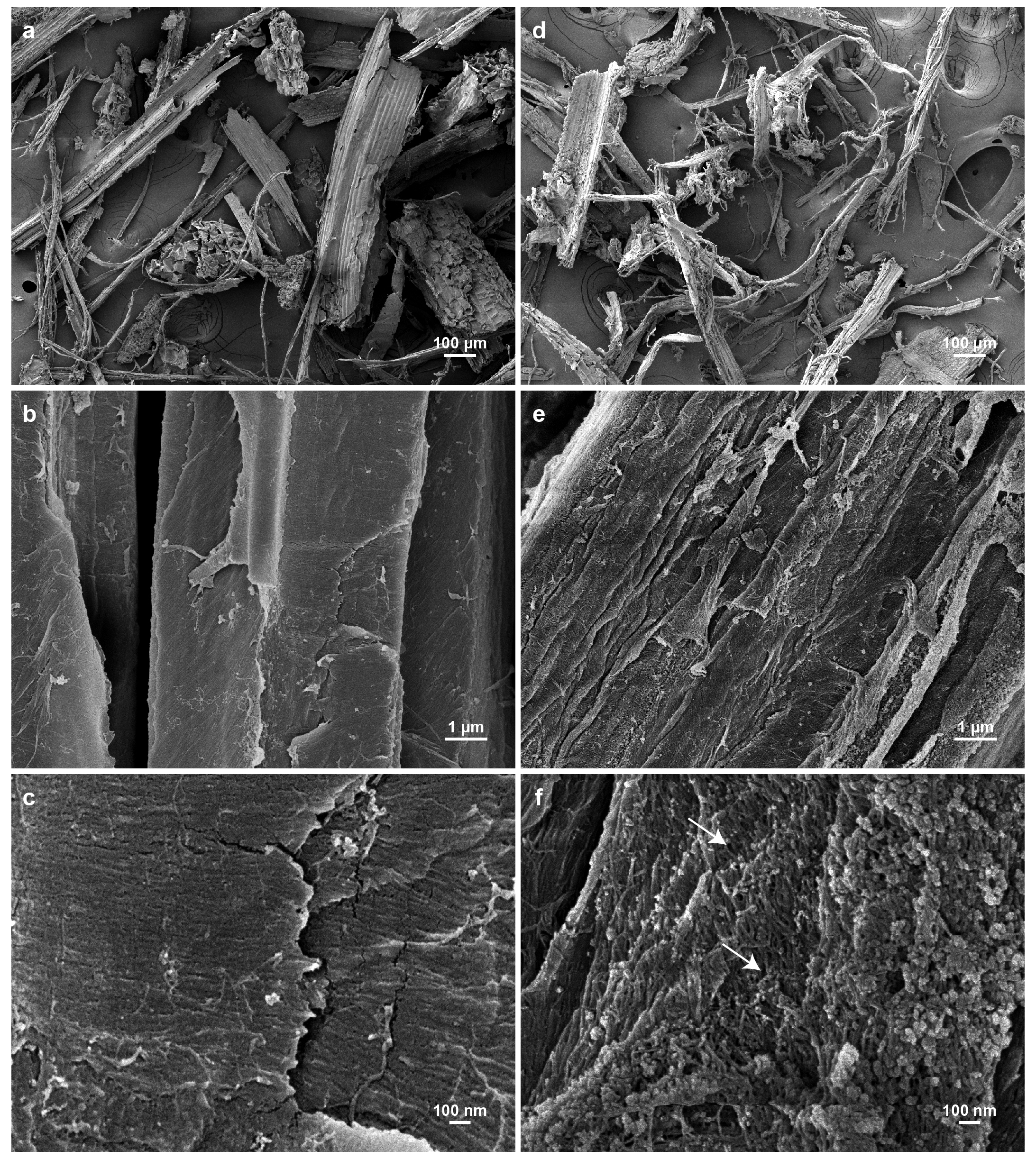

Supplement: Additional file 1: Figure S1 — FE-SEM photographs of rice straw powders showing the morphology of surface before (a-c) and after popping pretreatment (d-f). Note an increased in micropores (arrows) after popping pretreatment. [file 1754-6834-6-166-S1.jpeg]
