# Supplementary material for: Bioethanol production from rice straw by popping pretreatment
Source: Biotechnol Biofuels. 2013 Nov 29;6:166. doi: 10.1186/1754-6834-6-166 (PMC4176758; doi:10.1186/1754-6834-6-166)
Supplement: Additional file 2: Table S1 — ANOVA of the adjusted model of the response to enzymatic hydrolysis of pretreated rice straw. [file 1754-6834-6-166-S2.docx]

| **Source** | **Sum of squares** | **Degrees of freedom** | **Mean square** | **F value** | ***P*-value (Prob>F)** |
| --- | --- | --- | --- | --- | --- |
| **Model *x_1_*: cellulase loading**  ***x_2_*: xylanase loading**  ***x_1_ x_2_***  ***x_1_^2^***  ***x_2_^2^***  **Residual**  **Lack of Fit**  **Pure Error**  **Cor Total** | 6.43  3.39  0.026  0.031  2.97  0.36  0.32  0.31  0.010  6.74 | 5  1  1  1  1  1  5  3  2  10 | 1.29  3.39  0.026  0.031  2.97  0.36  0.063  0.10  5.033×10^-3^ | 20.36  53.63  0.42  0.49  47.13  5.67  20.23 | 0.0024  0.0007  0.5459  0.5167  0.0010  0.0630  0.0475 |
